# Disclosing the Decision to Decline Breast Screening and/or Breast Cancer Treatment Due to Concerns About Overdiagnosis and Overtreatment

**DOI:** 10.1111/hex.70468

**Published:** 2025-10-13

**Authors:** Shavez Jeffers, Alison Pilnick, Natalie Armstrong

**Affiliations:** ^1^ Diabetes Research Centre, Leicester General Hospital University of Leicester Leicester UK; ^2^ National Institute for Health and Care Research Applied Research Collaboration East Midlands Leicester UK; ^3^ School of Nursing and Public Health Manchester Metropolitan University Manchester UK; ^4^ School of Health & Medical Sciences, City St George's University of London London UK

## Abstract

**Background or Context:**

Overdiagnosis and overtreatment have been acknowledged as harms of the NHS Breast Screening Programme (BSP) due to the uncertainty around if, or how, non‐invasive and invasive cancers identified through screening will progress. Importance is therefore placed on encouraging individuals to make an informed choice about whether to participate in screening and any follow‐on interventions. Even though all screening programmes generally state explicitly that individuals should have the freedom to choose, research into wider cancer screening programmes shows how disclosing a decision to decline may be regarded as problematic by others. However, literature exploring experiences of disclosing the decision to decline breast screening or subsequent interventions within the UK context is limited.

**Objective:**

We explore women's experiences of disclosing the decision to decline screening, treatment and/or other recommended medical interventions after being invited to the NHS BSP, to understand how making the decision to decline breast screening and/or breast cancer treatment was received by others.

**Design:**

Semi‐structured interviews.

**Setting and Participants:**

Twenty women who had made the decision to decline screening, treatment and/or other interventions recommended after being invited to the NHS BSP were recruited through social media, online forums and word of mouth.

**Results:**

Some of the women discussed responses from their family and friends when disclosing their decision to decline and explained how they received supportive responses from some and negative responses from others. Difficulties in disclosing their intention to decline healthcare professionals were also discussed by some of the women. Receiving unsupportive responses meant that some of the women felt hesitant about how and where they disclosed their decision.

**Conclusions:**

To varying degrees, the findings revealed the burden of having to explain and account for the decision to decline and manage the potential reaction to this as not acceptable.

**Patient or Public Contribution:**

Before recruitment and data collection commenced, we sought feedback from an individual with lived experience in declining breast cancer screening and treatment due to concerns about overdiagnosis and overtreatment. This individual provided valuable insights on the study design and the most effective methods for recruiting participants from the targeted population. Additionally, a topic guide was developed for the semi‐structured interviews, which was then tested through a pilot interview with the same individual. The feedback from this pilot interview was instrumental in refining and improving the topic guide.

## 1 Introduction

### 1.1 Context

Screening is a widely utilised public health intervention, as it enables the identification and treatment of those who do not necessarily perceive themselves as being at risk and/or have no symptoms of disease [1, 2]. There are different types of screening programmes that are commonly delivered nationally within the United Kingdom, such as population‐based, targeted and stratified screening programmes [3], which differ in terms of whether they aim to identify risk factors or early stages of a condition/disease, whether they target individuals at higher risk of a condition/disease or whole populations, and whether screening tests are offered at regular intervals or dependent on an individual's decision or encounters with healthcare professionals [4].

Population‐based programmes invite a group of individuals identified from the whole population, defined by demographics such as sex or age, for screening [2] and have been implemented worldwide for a range of different conditions such as cancers, foetal conditions, newborn deficiencies, diabetic retinopathy, and abdominal aortic aneurysm [5]. To ensure that population‐based screening programmes are overall effective, implementation is often guided by a set of principles which include ensuring that the condition is an important problem, availability of treatment, suitability of screening test and the cost‐effectiveness of the screening programme overall [6]. These principles are considered in relation to both the population‐wide benefits and harms alongside implications for the individual [7]. However, due to advancements in screening equipment, diagnostic testing and treatments [8, 9], the rationale for certain population‐based screening programmes has come into question based on whether the benefits still outweigh the harms [10]. As a result of these ongoing debates, efforts have been made to ensure that individuals are supported and encouraged to make an informed choice through the dissemination of information regarding the potential harms as well as the benefits of all screening programmes [11]. The invocation of informed choice in this way implies that both making the decision to participate and not to participate should be seen as acceptable choices.

#### 1.1.1 The NHS BSP

The NHS Breast Screening Programme (NHS BSP) is a population‐based screening programme that is offered to women from the age of 50 to their 71st birthday, every 3 years. The aim of the NHS BSP is to reduce mortality from breast cancer by diagnosing cancer at an early stage when treatment is more successful [12]. This is achieved by sending women an invitation to attend a routine screening. Invitations are sent in the post and typically include either a pre‐booked timed appointment or an open invitation with instructions on how to book an appointment [13]. The invitation is also required to include an information leaflet explaining what participation in the programme involves, information about the benefits and potential harms of having routine breast screening, and explicitly stating that women should make an informed choice about whether to participate [14].

While breast screening with mammography can reduce mortality through early detection, it also carries potential harms such as false positives, false negatives, overdiagnosis and subsequent overtreatment, making the decision to participate complex [13]. False positives refer to a test result that indicates that a person has a specific disease or condition when they actually do not. In contrast, false negatives refer to a test result that indicates that a person does not have a specific disease or condition when the person actually does have the disease or condition [13]. Overdiagnosis can be defined as the identification of anomalies that might look like early diagnosis, but the things identified are not destined to cause symptoms or death [15]. Overdiagnosis is inherently a population‐level concept, meaning individuals cannot know with certainty whether their own diagnosis represents an overdiagnosed case [16]. Overdiagnosis can lead to overtreatment, which can be defined as unnecessary treatment for a condition that is not life‐threatening or would never cause any symptoms, and may lead to harmful side effects [17] such as physical harm from medical procedures [18], negative impact on well‐being [19] and reduced quality of life [20]. Rates of overdiagnosis have been identified as a potential harm of the NHS BSP due to the uncertainty around if, or how, non‐invasive and invasive cancers will progress. For example, Ductal Carcinoma In Situ (DCIS) is a non‐invasive breast cancer that is often detected during mammograms as part of the NHS BSP [21], as it is the earliest form of breast cancer [22]. DCIS is a precursor of invasive breast cancer [23], which means that while it may lead to invasive cancer, not all cases will progress [24].

#### 1.1.2 Difficulties Making the Decision to Decline

Due to the uncertainty around whether, or how, non‐invasive breast cancers will progress into invasive breast cancers, it is explicitly stated within the invitation to the NHS BSP that women have the freedom to choose whether to participate in the programme. However, there is limited literature focusing on the experiences of declining breast screening and/or breast cancer treatment. In addition, literature exploring experiences of declining cancer screening and/or cancer treatment more generally is mostly based on international studies, making it difficult to determine whether the findings are relevant across different countries. The organisation of different cancer screening programmes varies globally; for example, within some countries national cancer screening programmes are offered to individuals for free, such as Australia's national screening programme for breast cancer where women between 50 and 74 years old are invited for routine screening every 2 years [25]. Other countries require individuals to have health insurance or to pay a fee, for example, the United States offers a free national screening programme for breast and cervical cancer only to women with low incomes and little to no health insurance [26], which means that women who are not eligible for this programme are required to pay privately or seek services via their health insurance.

As well as international differences, there are also variations across cancer screening and cancer treatment in relation to cancer type. For example, within the United Kingdom, there are three NHS national screening programmes for cancers: those for breast, bowel and cervical cancer. All three of these cancer screening programmes are offered to those who are asymptomatic and invite individuals to routine screening tests [11], whereas other types of cancers, such as prostate cancer, are not offered as part of a national programme, but instead screening is offered in response to signs and symptoms [27]. Therefore, it is important to acknowledge that making an informed decision to participate (or not) in cancer screening and/or cancer treatment is affected by the availability and organisation of the screening being offered, which will also then impact treatment decisions.

Previous research exploring cancer screening and cancer treatment generally shows that deciding to decline may be regarded as problematic because those who choose to decline are potentially seen as lacking in judgement and careless, for reasons such as the assumption that screening is the optimal way to mitigate risk [28, 29]. However, research exploring the reasons and experiences of women who decline breast screening is limited. In the United Kingdom, trust in the NHS has traditionally played an important role, whereby research shows that service users trust the judgement, knowledge and expertise of health professionals to provide a competent service that meets their needs, and they trust the state to ensure equity in the allocation of public goods and services [30]. In other words, if a health intervention or service such as a cancer screening programme is offered by the NHS, the assumption for many people will be that benefits always outweigh the harms. Research exploring public attitudes towards cancer screening in the United Kingdom has found that there is a widespread belief that cancer screening is always a good idea, and this enthusiasm has arguably hampered attempts to inform the public of the limitations and possible harms of screening [31]. This wider context may make the experience of declining breast screening and/or breast cancer treatment and disclosing these decisions to others challenging.

Previous research in cancer screening and other clinical contexts has shown how disclosing the decision to decline can be met with unsupportive responses from healthcare professionals, friends and family. Davies et al. [19] explored experiences of people who self‐identified as having been overdiagnosed with thyroid cancer in the United States and found that some of their participants experienced difficult responses from healthcare professionals, friends and people online when they disclosed their decision to decline treatment. Similarly, Pickles et al. [20] explored the experiences of women across four different countries (the United Kingdom, the United States, Canada and Australia) who self‐identified as having a possible breast cancer overdiagnosis, and findings revealed that the women interviewed felt they had received little support and reassurance when discussing the possibility of overdiagnosis and/or overtreatment.

Overall, previous research on cancer screening and cancer treatment more generally has explored the experiences of individuals who have made the decision to decline because of concerns about overdiagnosis/overtreatment and has explored the experiences of individuals who self‐identified as overdiagnosed. Within those findings, the impact of support from family and friends and responses from healthcare professionals were discussed in relation to the impact of both positive and negative responses [19, 20]. However, there are no studies to the authors' knowledge exploring the experiences of UK women disclosing the decision to decline breast screening and/or breast cancer treatment due to the impact of possible overdiagnosis and/or overtreatment. Therefore, the aim of this paper is to explore women's experiences of disclosing the decision to decline screening, treatment and/or other recommended medical interventions after being invited to the NHS BSP, to understand how making the decision to decline breast screening and/or breast cancer treatment was received by others.

## 2 Materials and Methods

### 2.1 Data Collection

20 semi‐structured interviews were conducted with women who had declined one or more of the following after receiving an invitation to participate in the NHS BSP: (1) screening test, that is, mammogram; (2) further test, for example, biopsy and ultrasound, (3) treatment, for example, mastectomy, chemotherapy and radiotherapy and (4) any other medical intervention, for example, ongoing medication. Age criteria were not seen as necessary as the inclusion criteria included women that had been invited to the NHS Breast Screening which is sent out to eligible women from the age of 50 so anyone above this age was eligible to take part in this study.

Including women who declined screening, further investigation and/or treatment was appropriate given the study's aim to explore informed decision‐making across the NHS BSP pathway. Decisions about whether to participate in screening, undergo diagnostic procedures or accept treatment are often interconnected and based on similar concerns, such as perceptions of benefit, risk of overdiagnosis or scepticism about medical intervention. By including women who declined at different stages, the study captures the complexity of real‐world decision‐making and provides a more comprehensive understanding of how informed choices are made throughout the screening and treatment process.

Recruiting participants through NHS avenues was deemed inappropriate due to the difficulties of locating individuals who have declined health services. Therefore, the recruitment plan involved contacting third sector organisations that provided support for women's health nationally. Third sector organisations identified through an internet search of breast cancer support groups, menopause support groups and organisations that provided support for women's health nationally were contacted through phone calls and emails, as they were identified as potential gatekeepers to women who would fit the inclusion criteria. Organisations supporting women with menopause were contacted because individuals who experience menopause are likely to be at a similar age to those who are invited to the NHS BSP [14]. This approach was initially chosen as it can be one of the most far‐reaching and inexpensive ways of targeting a population [32]. In addition, gatekeepers can also include social networks and websites [33]. Therefore, the lead organisers of Facebook groups and forums that engaged with women about breast cancer, menopause and women's health overall were also contacted. However, the approach of contacting third sector organisations and leads of Facebook groups and forums was unsuccessful as there was a lack of participant interest and limited responses received from gatekeepers. This meant that amendments were made to the recruitment plan, which involved direct posting on social media sites such as Facebook and X (formerly known as Twitter), online forums such as Menopause Matters and Mumsnet, word of mouth and snowball sampling by asking participants to identify other potential participants, and sharing information about the project in an online overdiagnosis group. Further details are presented in Table 1.

**Table 1:** Characteristics of participants.

Pseudonym	Age	Recruitment avenue	What was accepted/declined along the NHS BSP pathway in chronological order	Time since last declined	Reason for declining	Health‐related occupation (self‐described)
Laura	Late 70s	Word of mouth	Accepted screening invitations, diagnosed with DCIS*** via NHS BSP, declined mastectomy, then accepted active monitoring/After 8–9 years, diagnosed with BC**, accepted mastectomy	9 years	Chose active monitoring instead due to lack of symptoms	Not known
Anna	Mid 50s	Overdiagnosis group	Accepted screening invitations, then decided to decline future screening invitations	3 years	Possibility of overdiagnosis and/or overtreatment	Geriatrician
Jodie	Late 50s	Twitter	Declined all screening invitations	5 months	Influenced by medical professionals	Medical writer/editor
Kathy	Early 50s	Word of mouth	Declined all screening invitations	2 years	Being at a low risk	No
Fern	Mid 60s	Twitter	Accepted screening invitations, diagnosed with BC** in the interval between screenings, accepted mastectomy, chemotherapy and radiotherapy, then declined adjunctive therapy and future screening invitations	8 years	Possibility of overdiagnosis and/or overtreatment	General Practitioner (GP)
Shannon	Late 50s	Overdiagnosis group	Declined all screening invitations	3–4 years	Being at a low risk/Possibility of overdiagnosis and/or overtreatment	General Practitioner (GP)
Donna	Late 50s	Twitter	Declined all screening invitations	8 years	Potential harms outweigh the benefits	General Practitioner (GP)
Hannah	Late 60s	Word of mouth	Accepted screening invitations, then decided to decline future screening invitations	2–3 years	Possibility of overdiagnosis and/or overtreatment	Author on women's health
Sam	Early 60s	Overdiagnosis group	Declined all screening invitations	12 years	Potential harms outweigh the benefits	Academic in women's health
Joanna	Early 60s	Word of mouth	Accepted screening invitations, diagnosed with BC via NHS BSP, accepted lumpectomy, mastectomy and biopsy, then declined future screening invitations	3 years	Possibility of overdiagnosis and/or overtreatment	Not known
Sylvester	Late 50s	Overdiagnosis group	Diagnosed with DCIS*** via NHS BSP, declined mastectomy, then accepted active monitoring/After 9 years, diagnosed with BC**, accepted mastectomy, chemotherapy and radiotherapy	9 years	Possibility of overdiagnosis and/or overtreatment	Not known
Julia	Early 60s	Overdiagnosis group	Declined all screening invitations	7 years	Possibility of overdiagnosis and/or overtreatment	Not known
Jess	Early 50s	Overdiagnosis group	Declined all screening invitations	6 weeks	Possibility of overdiagnosis and/or overtreatment	General Practitioner (GP)
Wendy	Late 40s	Overdiagnosis group	Declined all screening invitations before and after identified with the BRCA gene	2 years	Possibility of overdiagnosis and/or overtreatment	General Practitioner (GP)
Erica	*	Overdiagnosis group	Accepted screening invitations, then decided to decline future screening invitations	*	Possibility of overdiagnosis and/or overtreatment	General Practitioner (GP)
Natasha	Early 70s	Overdiagnosis group	Declined all screening invitations	A few months	Possibility of overdiagnosis and/or overtreatment	General Practitioner (GP)
Bridget	Early 60s	Breast Cancer charity forum	Accepted screening invitations, diagnosed with BC** via NHS BSP, radiotherapy, lumpectomy and drug therapy, then declined drug therapy due to side effects	6 months	Side effects of medication	Patient Advocate Volunteer at a breast cancer charity
Christine	*	Twitter	Identified with dense breasts via NHS BSP (pilot trial), then declined future screening invitations	A few months	Potential harms outweigh the benefits	Health Journalist
Tracey	*	Twitter	Declined all screening invitations	6 months	Possibility of overdiagnosis and/or overtreatment	General Practitioner (GP)
Emma	Early 50s	Word of mouth	Declined all screening invitations	6 months	Potential harms outweigh the benefits	Not known

* Missing data.

** BC = Breast Cancer.

*** DCIS = Ductal Carcinoma In Situ.

Those who responded and agreed to participate were sent a participant information sheet and asked to complete a consent form before being invited for an interview. Interviews were conducted by the first author online via Microsoft Teams or over the phone depending on the preference of the participant and lasted between 30 and 60 min. Before conducting the semi‐structured interviews, a topic guide was created to serve as a guide, in relation to the general order of the questions and the topics that were covered. The research was approved by the University of Leicester Ethics Sub‐Committee of Medicine and Biological Sciences.

### 2.2 Analysis

As the focus of this project was to explore lived experiences from individual perspectives, reflexive thematic analysis was chosen as the most suitable method for data analysis [34]. Preliminary analysis began simultaneously with conducting interviews, by taking field notes and reflecting on whether revisions needed to be made to the topic guide based on participant responses. Following this, all of the audio recordings were transcribed verbatim [35]. Once all the transcriptions were completed, computer‐assisted software (Nvivo) was used by the first author for the process of coding. Regular meetings including all authors guided rigorous theme development from those codes which were examined across the broader dataset to verify their relevance and accuracy and identify any deviant cases, leading to further refinement of the themes. These themes were then synthesised, and all authors reached a consensus on the final themes to capture common perspectives on declining breast screening and/or breast cancer treatment, with insights informed by existing social science and health screening literature on participation in screening programmes.

In the analysis that follows, quotations from participants are followed by pseudonyms and the phase of the screening or treatment process that was declined. This paper is from a wider study that explored the experiences of women who declined breast screening, breast cancer treatment and/or other recommended medical interventions after being invited to the NHS BSP. Concerns about overdiagnosis and overtreatment emerged as key influences in participants' decisions to decline invitations to the NHS BSP and/or breast cancer treatment. These concerns were expressed both explicitly—through direct reference to these concepts—and implicitly, through the ways participants described the reasoning and apprehensions underlying their choices. Details about how the women made their decision to decline are reported in a separate published article on this [36].

### 2.3 Reflexivity

Data collection and analysis were part of a PhD project, which was supervised by the second and third authors. At the time of data collection and analysis, the first author had never been invited to the NHS BSP due to age and had no family history of breast cancer. The academic background of the first author before starting this study included health studies (BA Hons), health psychology (MSc) and social science research (MSc). No previous academic or employment experiences were focused on breast screening or breast cancer treatment. The first author had no prior relationship with study participants.

Research design was also guided by an individual with lived experience in the area of declining due to potential overdiagnosis and/or overtreatment of breast cancer. Their feedback was sought to refine the study design and identify effective strategies for recruiting participants from the targeted population. Additionally, a topic guide (see Appendix 1) for the semi‐structured interviews was developed and tested through a pilot interview with the same individual. Insights from this pilot interview were used to further refine the topic guide.

## 3 Results

All women were asked about conversations in which they told others about their decision to decline; the experiences discussed included conversations with friends and family, responses from healthcare professionals, and scenarios where they did not feel comfortable disclosing their decision to decline and so had chosen not to do so.

### 3.1 Responses From Family and Friends

#### 3.1.1 Supportive Responses From Family

Some of the women reported a range of responses from different people with whom they had discussed their decisions. Some discussed how they explained their concerns about the harm of overdiagnosis and overtreatment to their family and subsequently felt reassured about their decision to decline:

My family were happy to go with what I felt was best and trusted my judgement and were very supportive.

(Laura (late 60s)—diagnosed with DCIS via NHS BSP, declined mastectomy, accepted active monitoring)

In the quote above, Laura discussed how she explained her choice to her family and how they were happy to support her with the decision that she made. Even though Laura did not explicitly state how her family demonstrated their support or whether they shared the same views about her decision to decline treatment, she did believe that her family ‘trusted her judgement’ to make the right decision for her.

Similarly, one of the women who had always declined invitations to the NHS BSP discussed how she felt that her friends and family had always supported her decisions regarding her health:

In terms of friends and family no, I never feel like anyone tries to pressure me to do anything really, you know, I'm supported in whatever my decisions are. Yeah, and yeah, and kind of validated really in, I feel like I feel like I've been validated really in my kind of decision making.

(Emma (early 50s)—declined all screening invitations)

In the quote above, Emma explains how she felt that her friends and family did not try to influence her decision but instead supported her decision, no matter what she decided to do. In some cases where women who had declined services discussed support, it was not clear whether/how much detail about the reason for declining had been shared with family and friends. However, in other cases, the women who made the decision to decline discussed how their family supported their decision after they had explained their reasons for making that choice:

Erm … my family were very supportive actually. Erm, they also struggled to understand how you could do such drastic surgery when you haven't got full blown cancer.

(Sylvester (late 50s)—diagnosed with DCIS via NHS BSP, declined mastectomy, accepted active monitoring)

Sylvester explained how she disclosed her decision to her family and reports that they were supportive because they shared the same concerns about the appropriateness of an invasive surgery for a non‐invasive early‐stage cancer—DCIS. It appeared that Sylvester was suggesting that her family would potentially have made similar decisions if they were in her position. This meant that Sylvester's family not only supported her decision to decline but also agreed with her reasons for making that choice.

#### 3.1.2 Negative Responses From Friends and Family

However, when disclosing their decision to decline, some of the women also discussed how they felt they had received unsupportive responses from friends and family. For example, Sylvester, who was quoted in the section above, explained that even though she felt supported by her family when disclosing her decision to decline to them, she also felt that she received a negative response from a close friend:

Erm, you know, a close friend said oh well why would you keep doing this and having, keep having to go for check‐ups (instead of having had some form of treatment). And obviously at that point you do get nervous don't you and that things might change and erm, why put yourself through it. Erm, but to me it was a no brainer.

(Sylvester (late 50s)—diagnosed with DCIS via NHS BSP, declined mastectomy, accepted active monitoring)

The response that Sylvester received from her friend made her question herself, ‘get nervous’, even though she didn't ultimately change her mind. Responses received from friends and family that involved highlighting concerns about risk were also discussed by some of the other women who made the decision to decline treatment. The women explained how these responses instilled worry about the possibility of their prognosis worsening—which is evident in Sylvester's quote above. However, all of the women who reported receiving responses which questioned their decisions made it clear that they did not change their minds about declining. Sylvester expresses the strength of her conviction in her decision‐making above by describing it as a ‘no‐brainer’.

Women who felt as though they were being perceived as irrational for declining screening or treatment reported that this was further exacerbated when there was a family history of cancer, as was the case for one of the women whose mother had died of breast cancer:

I mean there are the people who say, oh, you're mad, oh if my mother had died of cancer I would [be screened] blah blah blah.

(Hannah (late 60s)—declined all screening invitations)

Hannah's mother displayed symptoms in an interval between routine mammograms, and after having further tests, was diagnosed with an aggressive cancer. She reported reactions to her decisions as drawing on this familial knowledge to frame the irrationality of her decision to decline.

Other women also discussed how they received negative responses due to others suggesting that their decision to decline was irrational for other reasons, such as because it appeared to go against NHS recommendations. Some participants felt this assumption lay behind attempts to persuade them to change their minds and accept the medical intervention that was being offered, as Erica describes below:

Oh yes, my family thinks I'm mad [interviewer asks if she could explain why] They think that I'm being a bit extremist … you know my family is composed of intelligent lay people and their feeling is that if this was being promoted, particularly by the NHS erm then it must be worthwhile and I am probably missing the point.

(Erica (age unknown)—declined all screening invitations)

Erica explained how her family did not support her decision to decline screening, attributing this to their belief that she was being misguided or even extreme for declining an intervention that had been offered by the NHS, which was seen as a badge of its legitimacy. Other women also discussed how their friends responded to their decision by presenting the view that you should accept whatever healthcare is offered to you, especially when it is offered for free^1^:

Some friends are horrified and because they don't really understand and all they can see is, this is a free test why wouldn't you?…. They're just like no, it's what you do, you go for your smear test, you go for your mammogram, you go for your, whatever is offered you take it. Erm and, and that's fine. You know that's fine for them. But I think for me having done the research it was different. But yes, some friends were horrified.

(Christine (age unknown)—identified with dense breasts via NHS BSP (pilot trial), then declined future screening invitations)

Christine and some of the other women were met with unsupportive responses when they tried to disturb the assumption that there is no decision to be made about cancer screening, as it is always a good idea. Christine expressed that she respected her friends' views, but that, despite their reported efforts to persuade her, she was confident that declining screening based on the research that she had done was the best choice for her.

Overall, the quotes in this section demonstrate how some women reported a lack of support or even a challenge to their decision to decline. For some, this led to efforts made by friends and family to persuade them to accept breast screening and/or breast cancer treatment due to the belief that free health interventions that are offered by the NHS should always be accepted. Having considered responses from friends and family, we now turn to considering responses from healthcare professionals.

### 3.2 Difficulties Disclosing the Intention to Decline to Healthcare Professionals

When discussing the conversations that the women had with healthcare professionals, some of them referred to discussions regarding treatment options and physical examinations following a diagnosis of breast cancer and how they believed that the response they received from healthcare professionals made them feel patronised. For example, one of the women explained how, after having an abnormality found through participation in the NHS BSP, further tests revealed that she had DCIS in one of her breasts, and she was offered a mastectomy. She discussed her experience of disclosing her decision to decline a mastectomy within a clinical encounter, which involved a physical examination and a discussion about her treatment options:

Erm, the whole team at the hospital seemed to think that I was a problematic woman and a bit stupid…. It was the way that I was treated absolutely patronising they would not answer any of my questions.

(Laura (late 70s)—diagnosed with DCIS via NHS BSP, declined mastectomy, accepted active monitoring)

In the above quote, Laura discusses her experience of how she felt patronised because the healthcare professionals would not answer any of her questions. Even though Laura did not explicitly state the questions that she asked during her consultation, elsewhere in the interview, she did discuss how she was told that she had DCIS and that a mastectomy was the recommended treatment, but no further explanation of her diagnosis, the benefits and harms of a mastectomy, or alternative treatment options to a mastectomy was given.

It was also apparent that some of the women entered these interactions with the intention to decline at least some aspects of the recommended treatment and, therefore, from the women's perspective, it was a negative experience because disclosing their intention to decline was made difficult for them. For example, Fern explained that the oncologist she spoke to made her feel uncomfortable because she felt that she was being perceived as irrational:

When I was having treatment, erm, there was a discussion about whether I should have adjunctive therapy and, you know, I am an informed consumer, I read the papers, erm it was based on a non‐pre‐specified end point and very low erm, absolute improvement. And when I tried to discuss this with the oncologist, she just couldn't get it, she just said well why would you not want to have every possible treatment that might stop you dying of breast cancer. And when I said, well dying of breast cancer is not the worst thing that can happen to you she just looked as though I was clinically insane.

(Fern (mid 60s)—diagnosed with BC outside of the NHS BSP, accepted mastectomy, chemotherapy and radiotherapy, declined adjunctive therapy and future screening invitations)

In the quote above, Fern discussed a consultation that she had with her oncologist about a drug therapy that was offered in addition to the primary or initial therapy in an effort to maximise treatment effectiveness. Fern demonstrated her scientific knowledge of the effectiveness of the treatment to explain her concerns, but felt that the healthcare professional did not understand the point she was making, as the oncologist continued to attempt to persuade her using fatalistic language, that is, drawing attention to the risk of mortality.

Overall, the analysis in this section demonstrates how some women reported difficulties disclosing their intention to decline to healthcare professionals and how the responses they received made them feel as though they were being perceived as ‘problematic’ or ‘irrational’; this feeling was also mentioned by other women who discussed difficulties sharing their intention to decline with healthcare professionals. Having considered responses from healthcare professionals, we will now turn to considering situations where some of the women did not feel comfortable disclosing their decision to decline.

### 3.3 Avoiding Disclosing the Decision to Decline to Specific Groups of Women

Some of the women discussed situations where they were not comfortable disclosing their decision. One of the reasons why some women did not want to disclose their decision to specific people and/or groups was due to the negative responses that they had previously received. The anticipated judgement from others meant that some of the women did not want to tell particular groups of people about their decision to decline breast screening and/or treatment, so they self‐censored in anticipation. For example, one of the women explained her thoughts about potentially disclosing her decision to decline future screening invitations after going through breast cancer treatment to unknown members of a support group:

But I didn't think they would get my perspective and therefore I didn't go along to any kind of support groups…. I think I was frightened I'd encounter people who didn't understand and that was a, that the idea, the feeling of not being understood was er, that was painful. So, I didn't want to exacerbate that.

(Joanna (early 60s)—diagnosed with BC via NHS BSP, accepted lumpectomy, mastectomy and biopsy, declined future screening invitations)

Joanna explains how she avoided support groups due to fear of being misunderstood. Joanna's anticipated fear was a result of previous negative experiences that she had encountered when using online chatrooms. The unsupportive responses that she had received in the past from an online breast cancer community made her afraid that other breast cancer groups would respond to her experience, decision and views in the same way. Therefore, Joanna avoided support groups because she did not expect to receive support but instead was afraid of the psychological impact that the anticipated negative responses would cause her.

This expectation that support groups (formal or informal) have shared understandings of common experiences was also raised by other participants. For example, one of the women discussed her experience as part of a close group whose partners all served overseas in the armed forces. She did not disclose to the group that she had made the decision to decline all invitations to the NHS BSP, as she believed that she would receive a negative response:

I just wouldn't even dare start to discuss it with them because it was a quite a you know, it's almost like a club, isn't it? You know you sort of you all go and have your results so. So, I think there are people who you know, perhaps are more engaged with or they want to be engaged with this sort of, you know, with the services. Because it's you know, it's interesting to learn about your body and things and so I think there are some people who, who it would be difficult to, for me to have that conversation with.

(Jodie (late 50s)—declined all screening invitations)

Jodie explains how this group of women were close‐knit and shared the same views about learning about their bodies through healthcare, to the point of supporting each other by attending appointments together. Their engagements are framed by Jodie in terms of curiosity and free access. As with the formal support group that Joanna discussed, going for health screening was a shared experience that bonded the women together. Therefore, similarly to Joanna, for Jodie to disclose her decision to decline with them would have put her outside of their shared experience.

Overall, the quotes in the section above present how some of the women avoided disclosing their decision to decline in a range of contexts. However, their avoidance was for similar reasons: fear of being misunderstood and judged for their decision in a context where declining health services that are offered were not viewed as the norm.

## 4 Discussion

### 4.1 Summary of Findings

Overall, those who gained reassurance and support from their family and friends about their decision to decline expressed how they felt it confirmed that they were making the right decision for themselves. Gaining reassurance from those who knew them well appeared to be important for these women. One explanation as to why gaining reassurance appeared to be important could be that when family members express support and confidence in the individual's ability to make the right choice for them, it can lessen the burden of uncertainty in decision‐making [32]. In addition, making a decision that goes against the tide and is less common may be perceived as less socially acceptable. Therefore, it can be difficult to do if an individual feels alone in the choice that they have made. Receiving reassurance from those close to them and from those who know them well may have enabled these women to feel supported and alleviate some of the distress caused by uncertainty.

For those who highlighted negative experiences that they had with healthcare professionals within clinical encounters, there was a perception that disclosing their intention to decline was met with efforts to persuade them to reconsider their choice and change their minds. In addition, some of the women explained how they felt that they were viewed as ‘problematic’ or ‘irrational’, which is similar to previous research findings where those who chose to decline were potentially seen as lacking in judgement or careless [28, 29]. While some of the women interviewed reported that these persuasion attempts increased their anxiety or brought the focus onto the possible downsides of their choices, for all of the women, it did not diminish the belief that their decision to decline was the right choice for them. Therefore, this demonstrates how some of the women were accounting for how they stood firm in the face of attempted persuasion.

In specific contexts, some of the women discussed how they avoided disclosing their decision to decline for reasons such as not wanting to be judged by others. Fear of being judged could have potentially been a result of receiving unsupportive responses from friends, family and/or healthcare professionals previously. Alternatively, there is a possibility that some of the women feared being judged due to public attitudes towards cancer screening in the United Kingdom, as there is a widespread belief that cancer screening is always a good idea, potentially hampering attempts to inform the public of the limitations and possible harms of screening [31]. Whether or not previous negative experiences influenced anticipated judgement or awareness of the widespread public belief that screening is always a good idea, some of the women chose to navigate conversations about breast screening and/or treatment through avoidance.

Similarly to findings from Davies et al. [19] and Pickles et al. [20], this study demonstrates how those who made the decision to decline could be met with challenging responses when disclosing their decision to decline to friends, family and healthcare professionals. In addition, the finding that some of the women avoided disclosing their decision to decline to specific groups is potentially important and, to the authors' knowledge, has not been explored to date. This finding may suggest the potential presence of a double bind in relation to declining screening: that women may choose to not talk about their decision to decline as they recognise that it is a less common choice that may be negatively judged, but at the same time the fact that they do not talk about their decision to decline contributes to the perception that ‘everyone accepts screening’.

### 4.2 Limitations

A key strength of this study was the use of qualitative interviews, which enabled an in‐depth exploration of women's experiences with declining breast cancer screening and/or treatment. The study successfully recruited a population that has been under‐represented in previous research. However, the number of participants was small, and the study comprised a highly selected and unusual sample, as the majority had health‐related occupations. Recruiting women with health‐related occupations was not intentional; it is likely that the use of the word‐of‐mouth approach and sharing information about the project amongst an online overdiagnosis group contributed to the number of women who had health‐related occupations within the final sample.

As this was a small sample, it cannot be stated that thematic saturation was achieved. It should be noted that diagnoses and treatments reported were not verified, and they reflect the participants' perceptions and understandings. There were variations in time since declining screening/treatment (months to > 5 years), which may have also influenced perceptions. While some treatments may have been perceived by them as overtreatment, these findings should not be taken to suggest that the treatment offered was incorrect or of poor quality, as treatment recommendations may be made for many reasons, not all of which may be apparent.

### 4.3 Implications for Policy, Practice and Future Research

Within policy rhetoric, a solution to overdiagnosis and/or overtreatment is informed choice. However, some of the women who participated in this study discussed how disclosing their decision to decline was received negatively by healthcare professionals as well as friends and family, and how they were hesitant about disclosing their decision to groups of people who they believed would not understand their choice. These factors have implications for the women who make a choice to decline due to the burden of having to explain and account for their decision, and then manage the potential reaction to this as not acceptable. This may suggest that while the system officially recognises the importance of informed choice, by these women's accounts, it is not necessarily set up to facilitate it in the context of national screening programmes.

To better support informed choice, policy and practice need to shift beyond simply offering information toward fostering a cultural and professional environment in which a range of decisions—including declining—are respected. This could include additional training for healthcare professionals to address implicit biases and improve communication with patients who choose differently from expected norms. Future research could investigate how women from more diverse backgrounds experience the decision to decline, as this study included a relatively homogenous sample with health‐related occupations.

## 5 Conclusion

To varying degrees, the findings from this study revealed the problems which can ensue from being asked to make a choice when not all choices are treated as equivalent. The findings also revealed how important it was for women that the choice to decline was acknowledged as a reasonable and individual decision which suggests that in specific contexts women did not feel that others were supportive of their choice.

## Author Contributions

**Shavez Jeffers:** conceptualisation, investigation, writing – original draft, methodology, formal analysis, project administration, writing – review and editing. **Alison Pilnick:** funding acquisition, writing – review and editing, supervision, conceptualisation, formal analysis. **Natalie Armstrong:** funding acquisition, writing – review and editing, supervision, conceptualisation, formal analysis.

## Ethics Statement

This study was approved by the University of Leicester Ethics Sub‐Committee of Medicine and Biological Sciences (Project No. 23512).

## Consent

Informed consent was obtained from all participants.

## Conflicts of Interest

The authors declare no conflicts of interest.

## Reporting Guidelines

Standards for Reporting Qualitative Research (SRQR).

## Data Availability

The authors have nothing to report.

## Endnotes

1 Women with dense breasts have relatively high amounts of glandular tissue and fibrous connective tissue and relatively low amounts of fatty breast tissue [37], which makes mammogram results difficult to read, as abnormalities are harder to see [38].

## Appendix 1 Topic guide

**Navigating perceived overdiagnosis and overtreatment: a qualitative study—Topic guide**

**Welcome and introduction:**

Hello, my name is [Researcher's name] and I am a PhD student from the University of Leicester, can I confirm that I am speaking with ________.

Before we begin with the interview there are a couple of things that I would like to confirm with you?

Can you confirm that you have read the information sheet and the email consent statements.

Do you have any questions regarding the information that were emailed over to you?

Do you give your consent to those statement?

Are you happy with this interview being audio‐recorded?

No personal identifiable data will be recorded and a participant number will be allocated to you and sent you through email after the interview.

**Opening questions:**

Tell me about your experience with breast cancer services?

Can you tell me about your experience with the NHS breast cancer screening programme up until now?

Can you tell me what intervention you declined (screening/treatment/follow up tests/other?)

**The following questions refer to the intervention that you declined;**

Could you tell me about how you were offered the intervention?

How long ago has it been?

How do you feel about the way that it was offered to you?

How did you make your decision to decline? One‐off decision or definite decision?

Did you feel any pressure from anyone?

What sources of information guided your decision? Did you talk to anyone about it?

How do you feel about your decision now?

Do you feel differently now compared to when you first made the decision? What are the differences/similarities?

Did you tell anyone about your decision? Who did you tell? How did they respond? How did that make you feel?

Can you describe to me a conversation that you have had with a healthcare professional about your decision? Have you been prompted by healthcare professionals?

Since your decision, has your experience of interacting with healthcare professional changed?

Have you declined screening/treatment for breast cancer or any other service since then? Was that the first time you declined anything?

Have you considered or taken up any other services or avenues relating to breast cancer screening/treatment outside of NHS? For example, any alternative therapies.

Have you come across the idea of overdiagnosis? Could you explain what you know about it?

**Anything not covered?** Is there anything that we haven't covered in the interview that you think we should know or think about?

**Closing and thanks**—Are you still happy for you to use all the information provided?

You will receive an email, which will include your participant number and information about how to withdraw your contribution from the study.

Pseudonym? Own name?

Would you like to receive a summary of the findings? I will need to keep your contact details for this purpose

Is there anyone you know that might be interested in participating in this study?

Do you have any suggestions on how I could find more participants?

Thank you for your time and contribution.
